# Effects of Tai Chi on Mental Health in college students: a systematic review and meta-analysis of randomized controlled trials

**DOI:** 10.3389/fpubh.2026.1830203

**Published:** 2026-07-03

**Authors:** Kaiqiang Zhong, Xiujie Ma, Fangzhou He, Yifan Xu, Yue Ren, Liuqi Fu, Zhihao Chen, Guoqing Shen

**Affiliations:** 1 Gdansk University of Physical Education and Sport, Gdansk, Poland; 2 Chengdu Sport University, Chengdu, China; 3 Henan Sport University, Zhengzhou, China

**Keywords:** anxiety, college students, depression, mental health, meta-analysis, sleep quality, Tai Chi

## Abstract

**Objective:**

To systematically evaluate and quantitatively synthesize the effects of Tai Chi interventions on the mental health of college students.

**Methods:**

This study was conducted in accordance with the PRISMA 2020 guidelines for systematic reviews and meta-analyses and was registered in PROSPERO (CRD420261329121). PubMed, Web of Science, Scopus, Cochrane Library, EBSCO, CNKI, Wanfang Database, and the China Science and Technology Journal Database were systematically searched from database inception to February 1, 2026. Randomized controlled trials (RCTs) investigating the effects of Tai Chi interventions on the mental health of college students were included. Statistical analyses were performed using Review Manager 5.4. For continuous outcomes, standardized mean differences (SMDs) or mean differences (MDs) with 95% confidence intervals (CIs) were calculated. A random-effects model was used to pool the effect sizes. Between-study heterogeneity was assessed using Cochran’s Q test and the I*^2^* statistic. Sensitivity analyses and subgroup analyses were also conducted.

**Results:**

A total of 12 randomized controlled trials involving 1,057 college students were included (554 in the intervention group and 503 in the control group). The meta-analysis showed that Tai Chi intervention significantly reduced depression symptoms among college students (SMD = −0.67, 95% CI [−0.90, −0.43], *p* < 0.00001). Tai Chi also demonstrated significant effects in alleviating anxiety (SMD = −0.79, 95% CI [−1.42, −0.15], *p* = 0.02) and stress (SMD = −0.40, 95% CI [−0.65, −0.14], *p* = 0.002). In addition, Tai Chi significantly improved sleep quality among college students (MD = −2.14, 95% CI [−4.13, −0.15], *p* = 0.03), although substantial heterogeneity was observed across studies.

**Conclusion:**

The present meta-analysis provides quantitative evidence that Tai Chi interventions have beneficial effects on improving mental health among college students, particularly in reducing depression, anxiety, and stress. Although Tai Chi also appears to improve sleep quality, the relatively high heterogeneity across studies indicates that further high-quality randomized controlled trials are needed to confirm these findings and to determine optimal intervention protocols.

**Systematic review registration:**

PROSPERO: CRD420261329121, https://www.crd.york.ac.uk/PROSPERO/view/CRD420261329121.

## 1 Introduction

The university period represents a critical stage of psychological and physiological transition, during which students experience substantial academic demands, interpersonal challenges, and uncertainty regarding future career development. The university stage is often regarded as an important developmental phase of “emerging adulthood,” during which students must adapt to changes in learning environments, social roles, and increasing psychosocial demand (1, 2). Accordingly, this period is considered a high-risk stage for the development of psychological problems. As a result, university students are exposed to multiple sources of psychological stress. Large-scale surveys conducted in several countries have shown that mental health problems among university students are both common and widespread. For example, a large international survey conducted in 2019 reported that 15.4% of university students met the diagnostic criteria for at least one mental disorder within a 12-month period (3). Recent evidence suggests that mental health problems among university students have continued to increase in the post-pandemic era (4, 5). International studies have indicated that the prevalence of anxiety, depression, and sleep disturbances among university students is significantly higher than pre-pandemic levels. In some countries, the prevalence of anxiety and depressive symptoms among university students has exceeded 30% (6), while poor sleep quality, insufficient sleep duration, and circadian rhythm disturbances have also become increasingly common (7). These problems not only negatively affect academic performance and quality of life but are also associated with long-term mental health risks. Moreover, the COVID-19 pandemic has further increased the prevalence of depression and anxiety among young populations (8). Sleep disturbances are also strongly associated with psychological problems, and the relationship between sleep quality and mental health appears to be bidirectional (8, 9). Given the high prevalence of poor sleep quality among university students (10), this issue warrants particular attention. Therefore, developing effective interventions to improve mental health among university students has become an important priority in both public health and higher education.

Among the available approaches for improving mental health, pharmacological treatments have demonstrated clear efficacy in clinical populations. However, their accessibility and acceptance among university students are often limited due to concerns regarding side effects, cost, and stigma (11, 12). In recent years, exercise-based interventions have received increasing attention as non-pharmacological strategies for improving psychological well-being. A growing body of evidence suggests that various forms of physical activity can alleviate emotional symptoms and improve sleep and stress responses (13, 14). Nevertheless, many exercise programs require specialized facilities, equipment, or high levels of physical exertion, which may limit adherence among university students (15). Therefore, accessible and sustainable exercise interventions that are suitable for university settings remain an important area for exploration.

Tai Chi, a traditional Chinese mind–body exercise, has attracted considerable interest as a potential intervention for promoting mental health. Tai Chi is typically characterized by slow and continuous movements, controlled breathing, and focused attention, and is generally classified as a low-to-moderate-intensity exercise. These characteristics offer several advantages for university students, including ease of learning, low risk of injury, minimal requirements for space and equipment, and the integration of both aerobic exercise and mindfulness components (16). Compared with high-intensity or technically demanding forms of exercise, Tai Chi may be more suitable for promotion within university settings. Tai Chi is characterized by moderate exercise intensity, a relatively low learning threshold, and minimal requirements for equipment and facilities, which may enhance exercise adherence among university students (17). Furthermore, Tai Chi integrates physical activity, breathing regulation, and attentional control, and may therefore be particularly beneficial for students experiencing long-term psychological stress and emotional burden (18). These characteristics suggest that Tai Chi may serve not only as a form of physical exercise but also as a potentially effective strategy for psychological regulation and health promotion among university students. Evidence from randomized controlled trials (RCTs) supports the potential psychological benefits of Tai Chi. For example, Zhang et al. (19) reported that an 8-week “Bafa Wubu” Tai Chi intervention significantly reduced anxiety and depression scores among university students. Similarly, Wang et al. (20) found that Tai Chi training reduced anxiety levels and influenced theta oscillatory activity in the brain. In addition to these behavioral findings, previous studies have proposed several possible physiological and neurofunctional mechanisms underlying the mental health benefits of Tai Chi, including reduced activity of the hypothalamic–pituitary–adrenal (HPA) axis, improved autonomic balance, decreased cortisol levels, and altered functional connectivity in brain regions involved in emotional regulation (21).

To date, several systematic reviews and meta-analyses have examined the effects of Tai Chi on Mental Health-related outcomes. Previous evidence has suggested that Tai Chi may improve anxiety, depression, and sleep quality among older adults, patients with chronic diseases, and the general adult population (22, 23). However, most existing reviews have primarily focused on clinical or general adult populations, while evidence specifically targeting university students remains relatively limited. University students often experience elevated academic stress, irregular lifestyles, and unique psychosocial adaptation challenges, resulting in mental health needs that differ from those of other age groups (24). Although previous systematic reviews and meta-analyses have explored the psychological benefits of Tai Chi among university students or young populations, relatively few studies have systematically examined the potential influence of intervention dosage variables, such as training frequency, session duration, and total intervention period, on intervention outcomes, and the available evidence remains inconsistent (25, 26). Therefore, conducting a focused systematic review and meta-analysis targeting university students is warranted.

Despite these promising findings, several limitations remain in the existing body of evidence. First, many RCTs conducted among university students are pilot studies with relatively small sample sizes (19, 21), which may limit the reliability of individual study findings. Second, there is considerable variability in intervention dosage, including differences in training frequency, session duration, and total intervention period. Third, the measurement tools used to assess psychological outcomes vary across studies, which reduces the comparability of results. Taken together, the current evidence regarding the effects of Tai Chi on Mental Health among university students still lacks comprehensive integration, particularly with respect to quantitative analyses of different psychological outcomes and intervention dosage variables. This limitation may hinder the evidence-based implementation and promotion of Tai Chi interventions within university settings.

Therefore, a comprehensive synthesis of the available evidence is needed. The present study aimed to conduct a systematic review and meta-analysis of randomized controlled trials to evaluate the effects of Tai Chi on Mental Health outcomes among university students, including depression, anxiety, stress, and sleep quality. Specifically, this study aimed to:

- quantify the overall effect sizes of Tai Chi interventions on depression, anxiety, stress, and sleep quality among university students.
- examine the moderating effects of intervention dosage variables, including weekly training frequency, session duration, and total intervention duration.
- assess the risk of bias and potential publication bias of the included trials, thereby providing evidence-based recommendations for the implementation of Tai Chi programs in university settings.

## 2 Methods

This systematic review and meta-analysis was designed, conducted, and reported in accordance with the Preferred Reporting Items for Systematic Reviews and Meta-Analyses (PRISMA) 2020 guidelines. The study protocol was prospectively registered in PROSPERO (registration number: CRD420261329121).

### 2.1 Search strategy

Two researchers independently conducted a systematic search of the following Chinese and English electronic databases: PubMed, Web of Science, Scopus, Cochrane Library, EBSCO, CNKI, Wanfang Database, and the China Science and Technology Journal Database. All records from database inception to February 1, 2026 were included in the search.

During the search process, the Boolean operator “OR” was used to combine synonymous terms to broaden the search scope, whereas “AND” was used to combine different concepts to improve the relevance of the retrieved results. No restrictions were applied regarding publication year, sample size, or outcome measures.

In addition, the reference lists of all included studies and relevant systematic reviews were manually screened to identify potentially eligible studies that may have been missed during the database search (Table 1). Gray literature sources and trial registries were not included in the present review because the study aimed to synthesize peer-reviewed randomized controlled trials with sufficient methodological and outcome reporting quality.

**Table 1:** PubMed search strategy.

Search strategy
#1 “Tai Ji”[Mesh]
#2 “tai chi” OR taiji OR taijiquan OR “tai chi chuan” OR “tai ji quan” [All Fields]
#3 #1 OR #2
#4 “Students”[Mesh]
#5 students OR “college student*” OR “university student*” OR undergraduate* OR “graduate student*” [All Fields]
#6 #4 OR #5
#7 “Mental Health”[Mesh] OR “Psychological Phenomena”[Mesh]
#8 “mental health” OR depression OR anxiety OR stress OR sleep OR “sleep quality” OR emotion* OR mood OR “self-esteem” OR “self concept” OR “quality of life” [All Fields]
#9 #7 OR #8
#10 “randomized controlled trial” OR “controlled clinical trial” OR randomized OR randomised OR randomly OR trial [All Fields]
#11 #3 AND #6 AND #9 AND #10

### 2.2 Eligibility criteria

The PICO framework was defined as follows: Population (P): university students; Intervention (I): Tai Chi training; Comparison (C): conventional exercise, usual lifestyle activities, or no intervention; Outcome (O): mental health-related outcomes, including depression, anxiety, stress, and sleep quality. Accordingly, the present review aimed to evaluate whether Tai Chi interventions could improve mental health outcomes among university students compared with control conditions.

#### 2.2.1 Types of studies

Eligible studies were randomized controlled trials (RCTs) published in English or Chinese that investigated the effects of Tai Chi on the mental health of college students. Duplicate publications, reviews, systematic reviews, conference abstracts, dissertations, and non-randomized studies were excluded.

#### 2.2.2 Types of participants

The study population consisted of university students, with no restrictions on sex, age, or physical characteristics. Studies including healthy participants or individuals with mild to moderate psychological symptoms were eligible, provided that participants did not have exercise limitations or severe psychiatric disorders.

#### 2.2.3 Types of interventions

Participants in the intervention group received Tai Chi training, whereas those in the control group received conventional exercise, usual lifestyle activities, or no intervention. Studies in which Tai Chi was combined with other exercise modalities or therapeutic interventions were excluded.

#### 2.2.4 Types of outcome measures

Eligible studies were required to report at least one mental health-related outcome, including but not limited to depression, anxiety, stress, or sleep quality.

#### 2.2.5 Exclusion criteria

Studies were excluded if they met any of the following criteria:

1. Full text unavailable.
2. Duplicate publications.
3. Non-randomized controlled trials.
4. Inability to extract valid outcome data.
5. Interventions involving Tai Chi combined with other exercise or treatment methods.
6. Insufficient information or incomplete data.

### 2.3 Study selection and management

All retrieved records were imported into Zotero for literature management and duplicate removal. Two researchers independently screened the titles and abstracts of the identified studies, and the full texts of potentially eligible articles were subsequently retrieved for further assessment.

**Figure 1:**
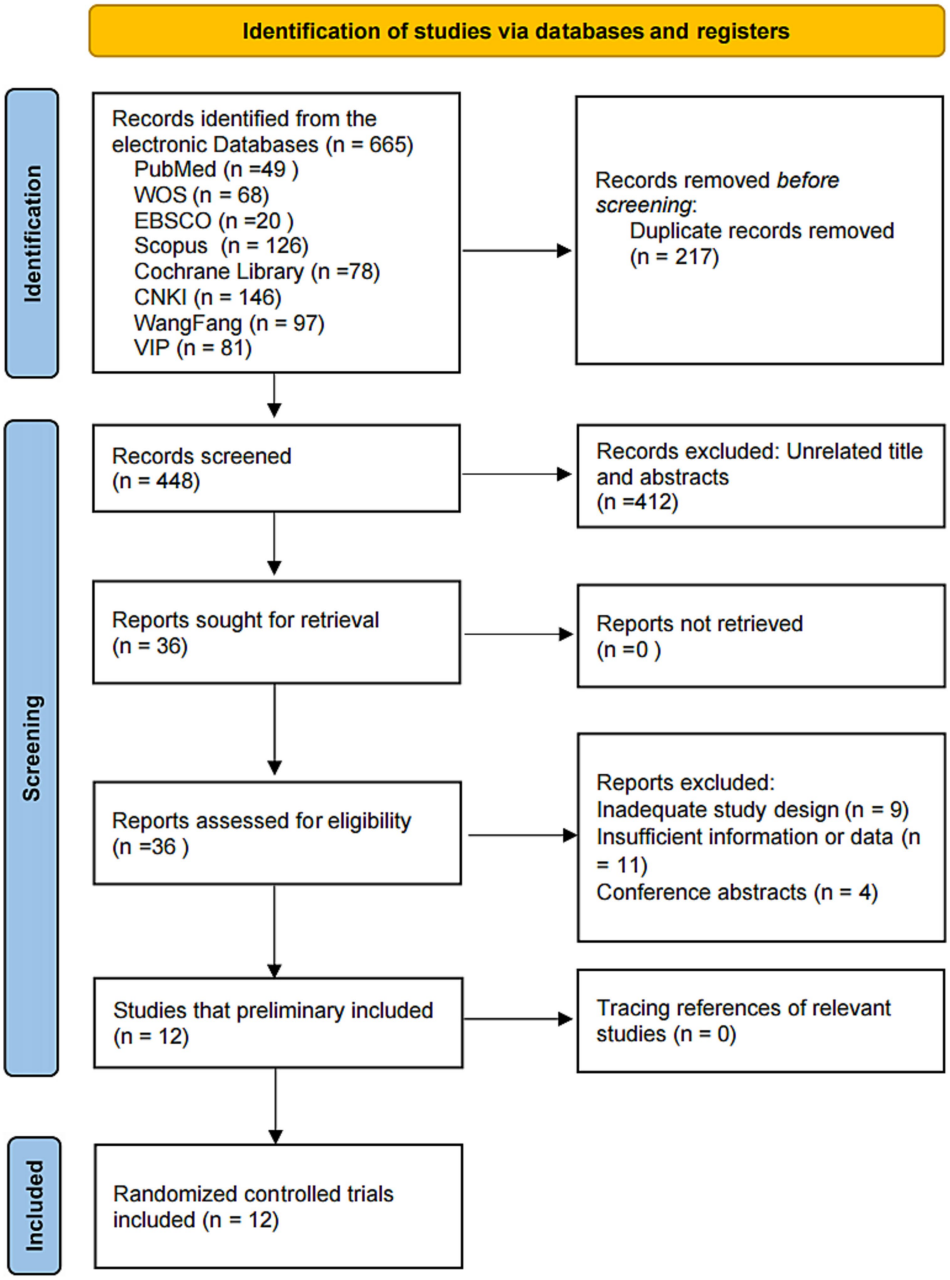
Flow diagram of literature search and selection process.

Any disagreements during the screening process were resolved through discussion. If consensus could not be reached, a third researcher was consulted to make the final decision. Inter-rater agreement during the study screening process was assessed using Cohen’s kappa coefficient, which indicated excellent agreement between the two reviewers (*κ* = 0.875, *p* < 0.001). The study selection process followed the PRISMA guidelines, and the detailed flow diagram is presented in Figure 1.

#### 2.3.1 Data extraction

Two researchers independently extracted data from the included studies using a predefined data extraction form. The extracted information included:

1. Basic study information: first author, publication year, study design, and country or region where the study was conducted.
2. Participant characteristics: health status, sample size, and mean age.
3. Intervention characteristics: type of Tai Chi, duration of each training session, training frequency, intervention period, and implementation method.
4. Control conditions: description of the intervention or lifestyle conditions in the control group.
5. Outcome measures and results data: primary and secondary outcomes related to mental health in college students, as well as statistical data required for the meta-analysis.

#### 2.3.2 Data verification

After the two reviewers completed independent data extraction, a third researcher independently verified the extracted data for accuracy and completeness. Any discrepancies identified during the verification process were resolved through discussion between the reviewers. If consensus could not be reached, the third researcher was consulted to make the final decision.

#### 2.3.3 Methodological quality and risk of bias assessment

The methodological quality and risk of bias of the included studies were independently assessed by two researchers. Randomized controlled trials were evaluated using the Cochrane Risk of Bias tool version 2.0 (RoB 2.0), which includes the following five domains:

1. Randomization process.
2. Deviations from intended interventions.
3. Missing outcome data.
4. Measurement of the outcome.
5. Selection of the reported result.

The risk of bias in each domain was rated as low risk, some concerns, or high risk, and an overall risk-of-bias judgment was subsequently determined. The assessment results were used only to describe the methodological quality of the included studies and were not used as criteria for excluding studies. Disagreements between the two reviewers were resolved through discussion. If necessary, a third researcher was consulted to reach a final decision.

### 2.4 Statistical analysis

Statistical analyses were conducted using Review Manager 5.4, and Stata 16 was used for supplementary analyses when necessary. For continuous outcomes, the post-intervention means and standard deviations reported in each study were extracted for the meta-analysis. When the same measurement scale was used across studies to assess a given outcome, the mean difference (MD) was calculated as the pooled effect size. When different measurement tools were used to assess the same outcome, the standardized mean difference (SMD) was used as the pooled effect size. All effect sizes were presented with 95% confidence intervals (95% CIs), and statistical significance was set at *p* < 0.05. Between-study heterogeneity was assessed using Cochran’s Q test and the I^2^ statistic. Given the anticipated clinical and methodological heterogeneity across studies, including differences in intervention protocols, intervention duration, participant characteristics, and outcome measures, a random-effects model was considered more appropriate for pooling effect sizes.

To evaluate the robustness of the results, leave-one-out sensitivity analyses were performed. When appropriate, subgroup analyses were conducted for the main outcomes based on intervention characteristics or types of measurement scales to explore potential sources of heterogeneity and better interpret variations in intervention effects across studies. When the number of studies included in a specific outcome was ≥10, publication bias was assessed visually using funnel plots and statistically using Egger’s regression test.

No data imputation procedures were performed. Studies with incomplete outcome data were excluded from the quantitative synthesis when the required statistical information could not be obtained from the published articles or through author contact.

## 3 Results

### 3.1 Study selection

A total of 665 records were identified through database searching, including PubMed (*n* = 49), Web of Science (*n* = 68), EBSCO (*n* = 20), Scopus (*n* = 126), Cochrane Library (*n* = 78), CNKI (*n* = 146), Wanfang (*n* = 97), and VIP (*n* = 81). After removing duplicates, 448 records remained for title and abstract screening. During this stage, 412 records were excluded because they were irrelevant to the research topic, leaving 36 articles for full-text assessment. Full texts were successfully retrieved for all identified articles (*n* = 36).

During the full-text review, 24 studies were excluded for the following reasons: study design not meeting the inclusion criteria (*n* = 9), insufficient information or unavailable data (*n* = 11), and conference abstracts (*n* = 4). In some studies excluded due to insufficient information, key outcome data were incompletely reported and additional data could not be obtained after contacting the authors; therefore, these studies were not included in the quantitative synthesis.

In addition, manual screening of the reference lists of included studies and relevant literature did not identify any additional eligible studies. Ultimately, 12 randomized controlled trials met the inclusion criteria and were included in the quantitative synthesis (Figure 1).

### 3.2 Characteristics of the included studies

The main characteristics of the included studies are summarized in Table 2. A total of 12 randomized controlled trials were included in this review, involving 1,057 university students, with 554 participants in the intervention group and 503 participants in the control group. The participants were primarily aged between 18 and 25 years and were all enrolled university students.

**Table 2:** Basic information of included literature.

Author (year)	Study design	Country	Participant characteristics	Group	Age, years (mean ± SD / range)	Sample size(n)	Intervention (type, frequency)	Intervention duration	Outcome
Chen et al. (30)	RCT	China	Female students with moderate depression	TG	NR	18	3 sessions/week, 60 min/session (24-form Tai Chi)	16 weeks	CES-D
				CG		18	No intervention		
Mao et al. (31)	RCT	China	NR	TG	18–20	52	3 sessions/week, 60 min/session (24-form Tai Chi)	18 weeks	SAS-20, SDS-20
				CG		52	No intervention		
Dinani et al. (32)	RCT	Iran	NR	TG	21.5 (mean)	32	3 sessions/week, 40 min/session (Yang-style Tai Chi)	8 weeks	DASS-42, EPQ
				CG		32	No intervention		
Zhou (33)	RCT	China	Female students with sleep disorders	TG	NR	30	3 sessions/week, 60 min/session (24-form Tai Chi)	16 weeks	PSQI
				CG		30	general physical activity		
Wang et al. (20)	RCT	China	Healthy students	TG	20.09 ± 0.29	22	3 sessions/week, 45 min/session (Tai Chi)	12 weeks	STAI
				CG	20.70 ± 0.56	23	Routine activities		
Zhang et al. (19)	RCT	China	Students with depression and anxiety	TG	24.2 ± 4.07	9	5 sessions/week, 60 min/session (Eight Methods and Five Steps Tai Chi)	8 weeks	SAS-20, SDS-20
				CG	22.50 ± 5.95	9	No intervention		
Zheng et al. (28)	RCT	China	Healthy students	TG	20.7 ± 1.1	95	5 sessions/week, 60 min/session (24-form Tai Chi)	12 weeks	GSES, SCL-90, PSS, SES, POMS, WHOQOL-BREF, PSQI
				CG	20.6 ± 1.2	103	No intervention		
Kong et al. (29)	RCT	China	Students with moderate depression and anxiety	TG	NR	30	5 sessions/week, 60 min/session (24-form Tai Chi)	8 weeks	SAS-20, SDS-20
				CG		27	No intervention		
Robert-McComb et al. (50)	RCT	USA	Healthy male students	TG	23 ± 5.1	9	2 sessions/week, 60 min/session (Eight Methods and Five Steps Tai Chi)	8 weeks	STAI
				CG	21.7 ± 1	11	No intervention		
Zhao (34)	RCT	China	NR	TG	19–24	40	3 sessions/week, 60 min/session (24-form Tai Chi)	12 weeks	SDS-20
				CG		20	No intervention		
Hua and Sun (35)	RCT	China	Healthy students	TG	20.38 ± 0.804	60	1 sessions/week, 80 min/session (24-form Tai Chi)	12 weeks	DASS-21, MASS
				CG	21.15 ± 1.040	20	No intervention		
Luo (36)	RCT	China	Students with sleep disorders	TG	18.58 ± 2.25	157	3 sessions/week, 30 min/session (24-form Tai Chi)	16 weeks	SAS-20, SDS-20, PSQI
				CG		158	No intervention		

RCT, Randomized Controlled Trial; NR, Not reported; TG, Tai Chi group; CG, Control group.

Across the included studies, Tai Chi interventions were compared with no intervention, usual daily activities, or general physical activity controls. The Tai Chi interventions included 24-form Tai Chi, Yang-style Tai Chi, and Bafa Wubu Tai Chi. The intervention duration ranged from 8 to 18 weeks, with 8 weeks (*n* = 4) and 12 weeks (*n* = 4) being the most common durations, followed by 16 weeks (*n* = 3) and 18 weeks (*n* = 1). The duration of each training session typically ranged from 40 to 80 min, with training frequencies ranging from 1 to 5 sessions per week.

Regarding outcome measures, the included studies primarily assessed mental health-related outcomes, including depression, anxiety, stress, sleep quality, self-efficacy, self-esteem, psychological symptoms, attention, mindfulness, and quality of life. All studies reported at least one mental health-related outcome.

Some studies included healthy university student samples, whereas others involved students experiencing mild to moderate psychological distress or sleep disturbances. Overall, there was considerable variability among the included studies in terms of participant characteristics, intervention protocols, and outcome measurement tools.

### 3.3 Methodological quality and risk of bias assessment

**Figure 2:**
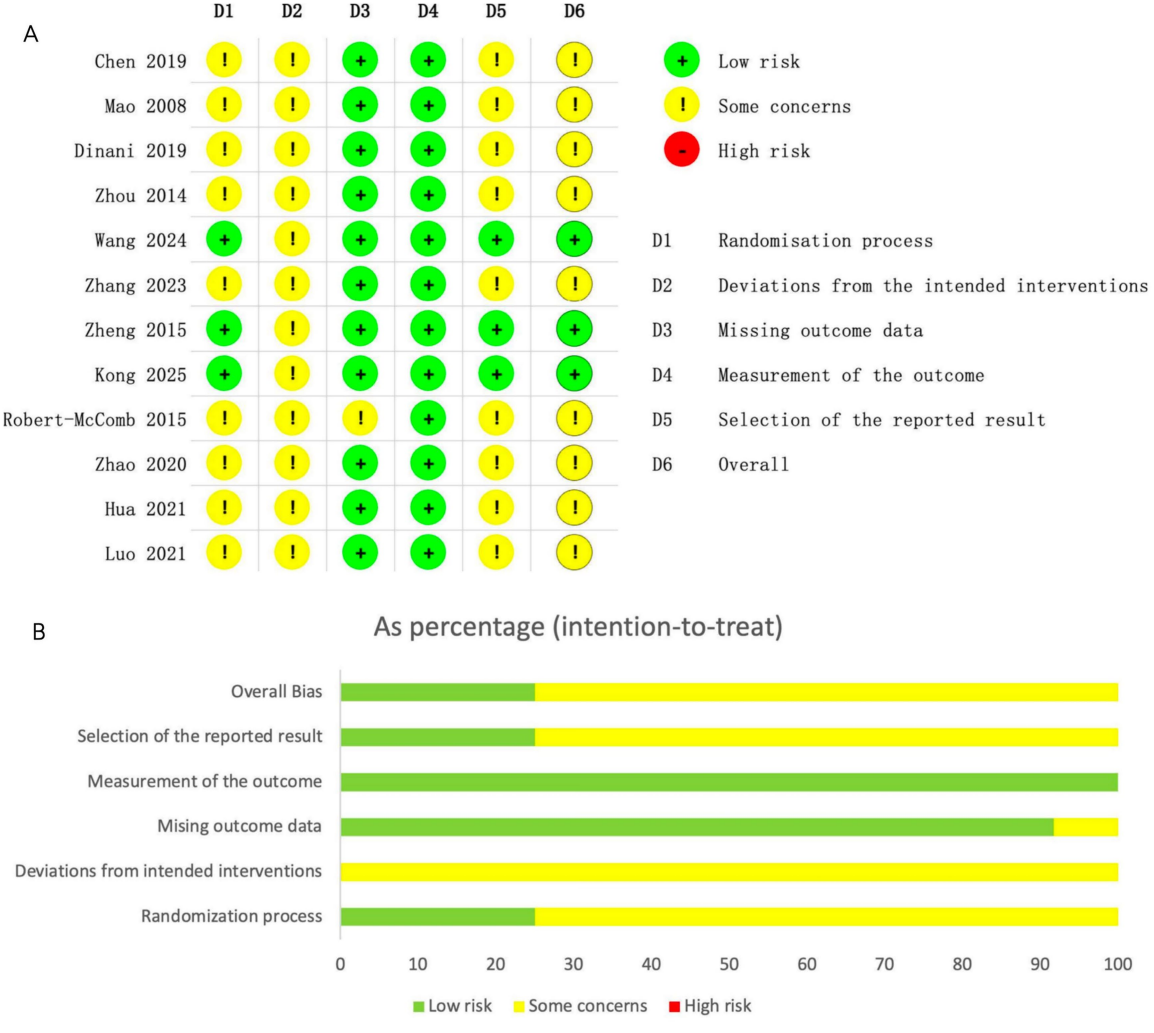
Risk of bias summary **(A)** and graph **(B)**.

The methodological quality of the included studies was assessed using the Cochrane Risk of Bias tool version 2 (RoB 2) (27). The detailed results are presented in Figures 2A,B.

Regarding the randomization process, all studies reported the use of random group allocation; however, the descriptions of random sequence generation and allocation concealment were often insufficient. Therefore, most studies in this domain were rated as “some concerns.” Wang (20), Zheng (28), and Kong (29) provided more detailed methodological information and were assessed as having low risk of bias in this domain.

For deviations from intended interventions, the overall assessment was relatively consistent. Because Tai Chi is a behavioral exercise intervention, blinding of participants and intervention providers is generally difficult to achieve, which is common in exercise intervention studies. Consequently, most studies did not clearly report blinding procedures, and this domain was frequently rated as “some concerns.” However, no clear evidence of systematic deviations from the intended interventions was identified.

Regarding missing outcome data, most studies reported complete outcome data or relatively low attrition rates. Studies such as Chen (30), Mao (31), Dinani (32), Zhou (33), Zhang (19), Zhao (34), Hua and Sun (35), and Luo (36) were therefore assessed as having low risk of bias in this domain. A few studies were rated as “some concerns” due to insufficient reporting of methods used to address missing data.

In terms of measurement of the outcome, all studies used standardized psychological assessment scales, indicating relatively standardized outcome assessment procedures. Consequently, this domain was generally rated as low risk of bias.

For selection of the reported results, most studies did not provide information on pre-registration or study protocols. Therefore, this domain was frequently rated as “some concerns.” However, no clear evidence of serious selective reporting was identified.

Overall, the risk-of-bias assessment indicated that most included studies were judged as having some concerns, while several studies were assessed as having low overall risk of bias.

### 3.4 Outcomes

#### 3.4.1 Effects of Tai Chi on depression among college students

A total of eight randomized controlled trials involving 734 participants (398 in the intervention group and 336 in the control group) were included in the analysis. A random-effects model was used to pool the effect sizes.

**Figure 3:**
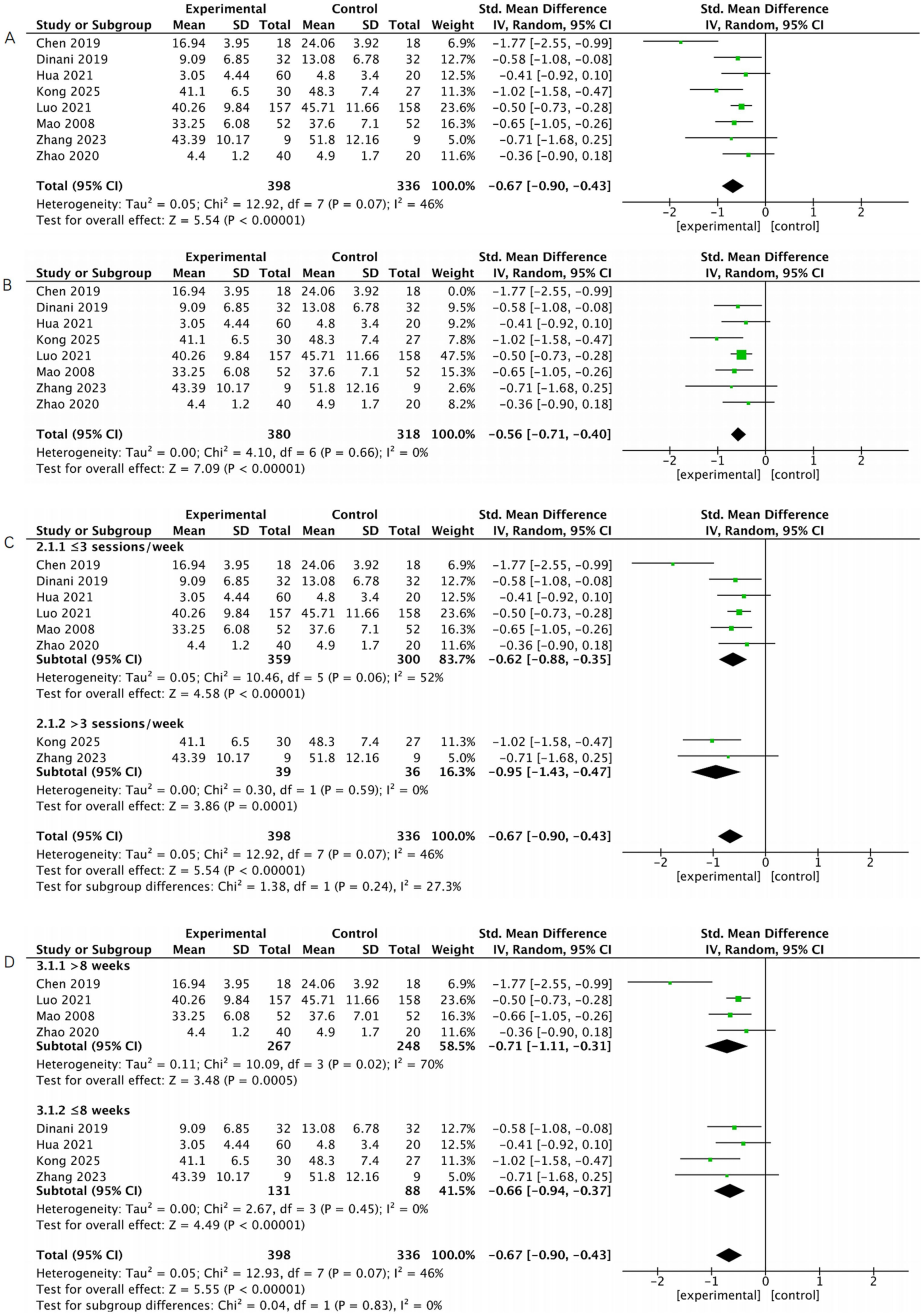
Forest plots of the effects of Tai Chi on depression among college students. **(A)** Overall meta-analysis. **(B)** Sensitivity analysis. **(C)** Subgroup analysis by training frequency. **(D)** Subgroup analysis by intervention duration.

The results showed that, compared with the control group, Tai Chi intervention significantly reduced depression symptoms among college students (SMD = −0.67, 95% CI [−0.90, −0.43], Z = 5.54, *p* < 0.00001). Moderate heterogeneity was observed among the studies (Tau^2^ = 0.05; Chi^2^ = 12.92, df = 7, *p* = 0.07; I^2^ = 46%; Figure 3A).

To evaluate the robustness of the results, a leave-one-out sensitivity analysis was performed. After excluding the study by Chen (30), the pooled effect size remained statistically significant (SMD = −0.56, 95% CI [−0.71, −0.40], Z = 7.09, *p* < 0.00001), and heterogeneity decreased to I^2^ = 0% (Chi^2^ = 4.10, df = 6, *p* = 0.66; Figure 3B).

Subgroup analyses were conducted based on training frequency. Studies with ≤3 sessions per week showed a significant effect (SMD = −0.62, 95% CI [−0.88, −0.35], Z = 4.58, *p* < 0.00001), with moderate heterogeneity (I^2^ = 52%). Studies with >3 sessions per week also showed a significant effect with a larger effect size (SMD = −0.95, 95% CI [−1.43, −0.47], Z = 3.86, *p* = 0.0001), and no heterogeneity was observed (I^2^ = 0%). However, no significant difference between subgroups was identified (Chi^2^ = 1.38, df = 1, *p* = 0.24; Figure 3C).

Subgroup analysis based on intervention duration showed that studies with intervention periods >8 weeks had a pooled effect size of SMD = −0.71 (95% CI [−1.11, −0.31], Z = 3.48, *p* = 0.0005), with substantial heterogeneity (I^2^ = 70%). For studies with intervention periods ≤8 weeks, the pooled effect size was SMD = −0.66 (95% CI [−0.94, −0.37], Z = 4.49, *p* < 0.00001), with no heterogeneity (I^2^ = 0%). The difference between subgroups was not statistically significant (Chi^2^ = 0.04, df = 1, *p* = 0.83; Figure 3D).

#### 3.4.2 Effects of Tai Chi on anxiety among college students

A total of eight randomized controlled trials involving 703 participants (371 in the intervention group and 332 in the control group) were included in the analysis. A random-effects model was used to pool the effect sizes.

**Figure 4:**
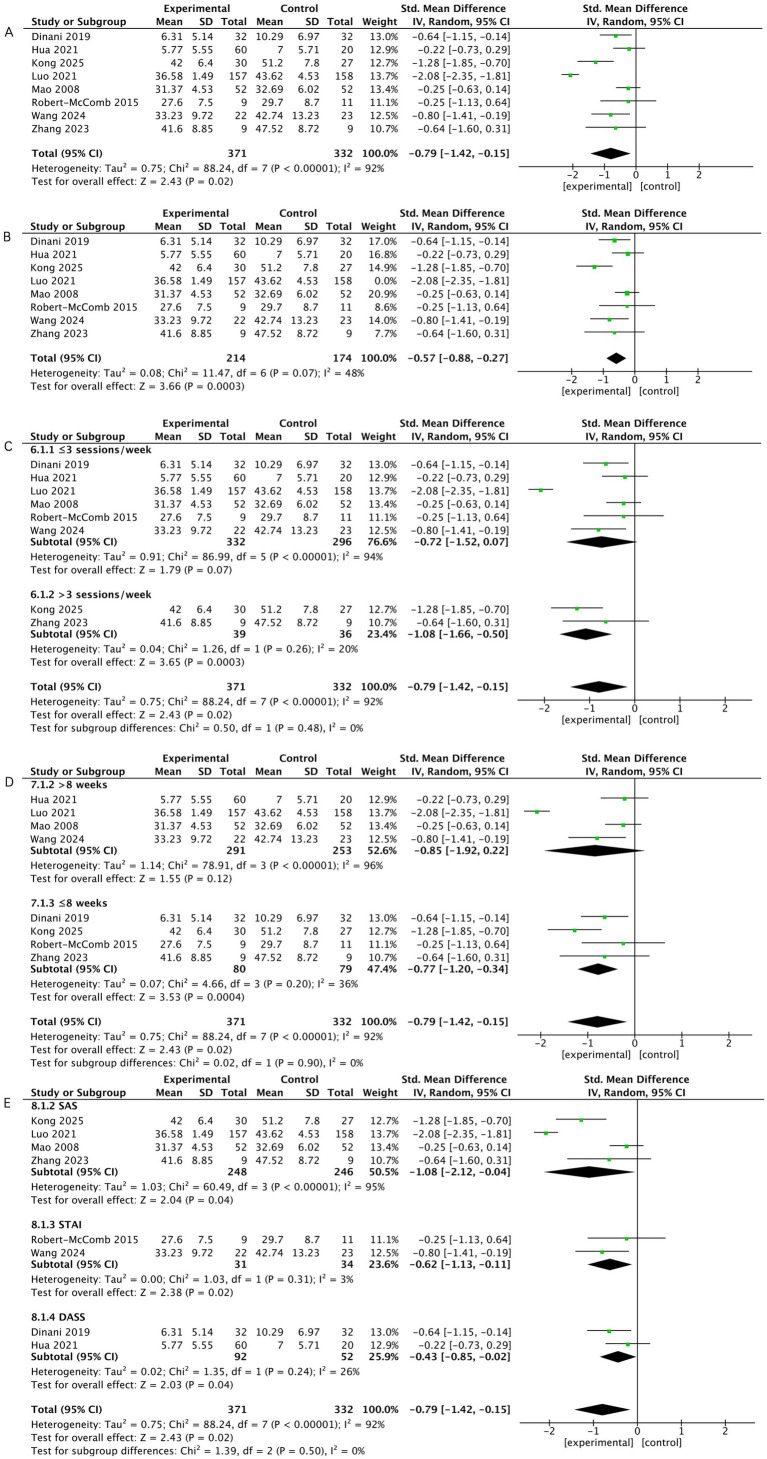
Forest plots of the effects of Tai Chi on anxiety among college students. **(A)** Overall meta-analysis. **(B)** Leave-one-out sensitivity analysis. **(C)** Subgroup analysis by training frequency. **(D)** Subgroup analysis by intervention duration. **(E)** Subgroup analysis by measurement tools.

The results showed that, compared with the control group, Tai Chi intervention significantly reduced anxiety symptoms among college students (SMD = −0.79, 95% CI [−1.42, −0.15], Z = 2.43, *p* = 0.02). High heterogeneity was observed among the studies (Tau^2^ = 0.75; Chi^2^ = 88.24, df = 7, *p* < 0.00001; I^2^ = 92%; Figure 4A).

To assess the robustness of the results, a leave-one-out sensitivity analysis was conducted. After excluding the study by Luo (36), the pooled effect remained statistically significant (SMD = −0.57, 95% CI [−0.88, −0.27], Z = 3.66, *p* = 0.0003), and heterogeneity decreased from 92 to 48% (Tau^2^ = 0.08; Chi^2^ = 11.47, df = 6, *p* = 0.07; Figure 4B).

Sequential exclusion analysis further showed that removing any of the remaining studies resulted in I^2^ values ranging from 91 to 93%, indicating that Luo (36) contributed substantially to the observed heterogeneity, while the overall intervention effect remained stable (Table 3).

**Table 3:** Sensitivity analysis using the leave-one-out method for anxiety.

Study removed	Pooled effect (SMD)	95% CI	I^2^	*p*-value	Conclusion
Dinani et al. (32)	−0.81	[−1.53, −0.08]	93%	0.03	Stable
Hua and Sun (35)	−0.87	[−1.56, −0.19]	92%	0.01	Stable
Kong et al. (29)	−0.71	[−1.44, 0.01]	93%	0.05	Borderline
(36)	−0.57	[−0.88, −0.27]	48%	0.0003	Heterogeneity reduced
Mao et al. (31)	−0.87	[−1.54, −0.20]	91%	0.01	Stable
Robert-McComb et al. (50)	−0.78	[−1.50, −0.07]	93%	0.03	Stable
Wang et al. (20)	−0.81	[−1.49, −0.12]	93%	0.02	Stable
Zhang et al. (19)	−0.86	[−1.54, −0.18]	93%	0.01	Stable

Subgroup analysis based on training frequency indicated that studies with ≤3 sessions per week yielded a pooled effect size of SMD = −0.72 (95% CI [−1.52, 0.07], Z = 1.79, *p* = 0.07), which was not statistically significant, with high heterogeneity (I^2^ = 94%). In contrast, studies with >3 sessions per week demonstrated a significant intervention effect (SMD = −1.08, 95% CI [−1.66, −0.50], Z = 3.65, *p* = 0.0003) with low heterogeneity (I^2^ = 20%). However, the difference between subgroups was not statistically significant (Chi^2^ = 0.50, df = 1, *p* = 0.48; Figure 4C).

Subgroup analysis based on intervention duration showed that studies with intervention periods >8 weeks had a pooled effect size of SMD = −0.85 (95% CI [−1.92, 0.22], Z = 1.55, *p* = 0.12), which was not statistically significant and showed high heterogeneity (I^2^ = 96%). Studies with intervention periods ≤8 weeks demonstrated a significant effect (SMD = −0.77, 95% CI [−1.20, −0.34], Z = 3.53, *p* = 0.0004) with moderate heterogeneity (I^2^ = 36%). The difference between subgroups was not statistically significant (Chi^2^ = 0.02, df = 1, *p* = 0.90; Figure 4D).

Subgroup analysis based on measurement tools indicated that studies using the SAS scale showed a pooled effect size of SMD = −1.08 (95% CI [−2.12, −0.04], Z = 2.04, *p* = 0.04), with high heterogeneity (I^2^ = 95%). Studies using the STAI scale showed a pooled effect size of SMD = −0.62 (95% CI [−1.13, −0.11], Z = 2.38, *p* = 0.02), with low heterogeneity (I^2^ = 3%). Studies using the DASS scale showed a pooled effect size of SMD = −0.43 (95% CI [−0.85, −0.02], Z = 2.03, *p* = 0.04), with moderate heterogeneity (I^2^ = 26%). The differences between subgroups were not statistically significant (Chi^2^ = 1.39, df = 2, *p* = 0.50; Figure 4E).

#### 3.4.3 Effects of Tai Chi on stress among college students

**Figure 5:**
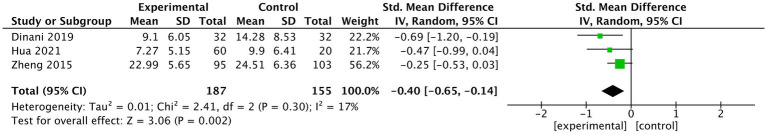
Forest plots of the effects of Tai Chi on stress among college students.

A total of three randomized controlled trials involving 342 participants (187 in the intervention group and 155 in the control group) were included in the analysis. A random-effects model was used to pool the effect sizes. The results showed that Tai Chi intervention significantly reduced stress symptoms among college students (SMD = −0.40, 95% CI [−0.65, −0.14], Z = 3.06, *p* = 0.002). Low heterogeneity was observed among the studies (Tau^2^ = 0.01; Chi^2^ = 2.41, df = 2, *p* = 0.30; I^2^ = 17%), indicating relatively consistent findings across studies (Figure 5). Due to the limited number of included studies and the low heterogeneity, further subgroup or sensitivity analyses were not conducted.

#### 3.4.4 Effects of Tai Chi on sleep quality among college students

**Figure 6:**
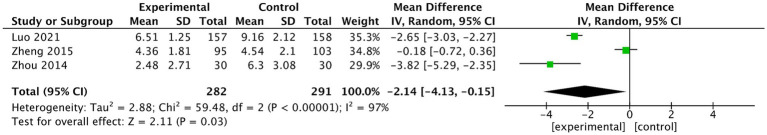
Forest plot of the effects of Tai Chi on sleep quality among college students.

A total of three randomized controlled trials involving 573 participants were included in the analysis, with 282 participants in the intervention group and 291 in the control group. A random-effects model was used to pool the effect sizes. The results showed that Tai Chi intervention significantly improved sleep quality among college students (MD = −2.14, 95% CI [−4.13, −0.15], *p* = 0.03; Figure 6). The negative effect size indicates lower sleep quality scores in the intervention group, reflecting improved sleep quality. However, substantial heterogeneity was observed among the studies (Tau^2^ = 2.88; Chi^2^ = 59.48, df = 2, *p* < 0.00001; I^2^ = 97%).

Formal assessment of publication bias using funnel plots and Egger’s regression test was not performed because fewer than 10 studies were included in each outcome analysis, which may reduce the reliability and interpretability of these methods.

### 3.5 Certainty of evidence assessment

The certainty of evidence for each outcome was evaluated using the GRADE approach. The certainty of evidence varied across outcomes, ranging from moderate to very low (Table 4).

**Table 4:** GRADE assessment of certainty of evidence across outcomes.

Outcome	Studies (n)	Participants (n)	Pooled effect (95% CI)	Heterogeneity	Certainty of evidence	Main reasons for downgrading
Depression	8	734	SMD = −0.67 (−0.90 to −0.43)	I^2^ = 46%	Moderate	Risk of bias, moderate inconsistency
Anxiety	8	703	SMD = −0.79 (−1.42 to −0.15)	I^2^ = 92%	Low	Risk of bias, serious inconsistency
Stress	3	342	SMD = −0.40 (−0.65 to −0.14)	I^2^ = 17%	Moderate	Risk of bias, limited number of studies
Sleep quality	3	573	MD = −2.14 (−4.13 to −0.15)	I^2^ = 97%	Very low	Serious inconsistency, imprecision, limited number of studies

The certainty of evidence for depression was rated as moderate because of some concerns regarding risk of bias and moderate inconsistency across studies. The certainty of evidence for anxiety was rated as low owing to substantial between-study heterogeneity (I^2^ = 92%) and concerns regarding risk of bias. The certainty of evidence for stress was rated as moderate because, although the pooled results were relatively consistent, the limited number of included studies and some concerns regarding risk of bias reduced confidence in the estimates. The certainty of evidence for sleep quality was rated as very low because of serious inconsistency across studies (I^2^ = 97%), imprecision, and the limited number of included studies.

## 4 Discussion

### 4.1 Effects of Tai Chi on Mental Health indicators among college students

The results of this meta-analysis indicate that Tai Chi interventions have significant beneficial effects on negative emotional symptoms among college students. The reductions in both depression and anxiety symptoms in the Tai Chi group were statistically significant, with moderate to large effect sizes (|SMD| = 0.67–0.79), which are generally consistent with findings from previous studies (18, 26, 37). In comparison, the improvement in perceived stress demonstrated a moderate effect size with low heterogeneity, indicating relatively consistent findings across studies. These results are also in line with previous research suggesting that Tai Chi can effectively reduce perceived stress in different populations (38, 39). Regarding sleep quality, the three trials included in this study showed that sleep scores in the Tai Chi group were lower than those in the control group, indicating improved sleep quality. However, very high heterogeneity was observed among the studies (I^2^ = 97%). Given the limited number of included studies and the substantial variability in results, the current evidence only suggests a potential improvement in sleep quality, and the robustness of this effect requires further verification through larger and more rigorous trials. Overall, the findings of the present study suggest that Tai Chi has consistent positive effects in reducing depression, anxiety, and stress among college students, whereas the evidence regarding its effects on sleep quality remains inconclusive.

### 4.2 Mechanisms of action

As a typical mind–body exercise, the beneficial effects of Tai Chi may be mediated through multiple physiological and psychological mechanisms (40, 41). From a physiological perspective, Tai Chi involves slow and rhythmic movements combined with breathing regulation, which may reduce sympathetic nervous system activity and enhance parasympathetic function, thereby alleviating stress responses (42). Previous research has shown that after 12 weeks of Tai Chi practice, salivary cortisol levels among university students decreased significantly, suggesting suppression of hypothalamic–pituitary–adrenal (HPA) axis activity (43).

Moreover, Tai Chi is a low- to moderate-intensity aerobic exercise that can improve cardiorespiratory fitness, flexibility, and balance, thereby contributing to overall physical health (44). Regular physical activity may also enhance self-efficacy and social interaction, which can indirectly promote improvements in emotional well-being.

From a psychological perspective, Tai Chi emphasizes focused attention and mindful movement. During practice, individuals concentrate on their body movements and breathing patterns, which resemble the core principles of mindfulness training. This process may help interrupt ruminative thought patterns and enhance self-regulation and psychological resilience (41). Neuroimaging evidence further supports this explanation. Several fMRI studies have demonstrated that long-term Tai Chi practice can induce structural and functional changes in the prefrontal cortex, a key brain region involved in executive control and emotional regulation (45, 46). Improved prefrontal cortex function may therefore contribute to better regulation of negative emotions. Taken together, Tai Chi may alleviate depression, anxiety, and stress through a combination of neuroendocrine regulation, enhancement of brain emotional regulation networks, and training in breathing and body awareness.

### 4.3 Feasibility and practical significance

Tai Chi is characterized by low intensity, high safety, and low cost, making it particularly suitable for implementation among university students. Unlike exercises that require complex equipment or high-intensity training, Tai Chi can be practiced in relatively small indoor or outdoor spaces without specialized equipment. In addition, the learning process is progressive, allowing beginners to gradually master the movements and maintain adherence (47). Empirical studies have demonstrated that structured Tai Chi programs designed for university students experiencing high levels of stress are both feasible and well accepted. For example, a 16-week intervention study involving university students with high perceived stress reported that the Tai Chi group showed significantly greater improvements than the control group across multiple indicators of physical and mental health. Participants reported not only reduced stress levels, but also improvements in sleep quality and physical fitness (39). Furthermore, qualitative studies suggest that many university students perceive Tai Chi as a promising strategy for psychological regulation, as it promotes the integration of physical and mental well-being while also facilitating social interaction (48, 49). Collectively, these findings indicate that Tai Chi interventions are not only scientifically effective, but also practical and feasible for implementation within university settings, highlighting their potential value as a campus-based health promotion strategy.

### 4.4 Study limitations and future perspectives

Several limitations of this study should be acknowledged. First, the number of included studies was relatively limited, particularly for outcomes related to stress and sleep, which may have reduced the statistical power of the analyses. Second, some studies provided insufficient methodological details regarding randomization procedures, allocation concealment, and blinding, indicating potential risks of bias. Furthermore, Tai Chi interventions were not standardized across studies, and variations in training style, frequency, and intervention duration may have contributed to the observed heterogeneity. Although subgroup analyses suggested that training frequency and intervention duration might influence effect sizes, the limited number of studies and the absence of statistically significant subgroup differences prevented the identification of an optimal intervention dosage.

Future research should focus on several directions. First, large-scale, multicenter randomized controlled trials with rigorous methodological designs are needed, with clearer reporting of bias-control procedures such as randomization and blinding. Second, intervention protocols should be described in greater detail, including the specific Tai Chi forms, training frequency, and session duration. In addition, future studies should incorporate a broader range of health indicators and long-term follow-up assessments to evaluate the sustained effects of Tai Chi interventions. Moreover, it may be valuable to explore the differential effects of various Tai Chi practices, such as instrument-assisted Tai Chi or push-hands, on mental health outcomes. These efforts will help refine intervention strategies and maximize the potential benefits of Tai Chi for improving the physical and mental health of university students.

## 5 Conclusion

The findings of this meta-analysis indicate that Tai Chi interventions have moderate to large beneficial effects in reducing depression, anxiety, and stress among university students, whereas the evidence regarding sleep quality suggests a potential but inconclusive improvement. Although intervention outcomes may be influenced by training dosage and study quality, the current evidence suggests that Tai Chi is a safe, feasible, and accessible approach for promoting mental health in university settings. Given these advantages, Tai Chi could be incorporated into university-based health programs, such as physical activity electives or student stress-management programs. Future research should include larger and more rigorously designed randomized controlled trials to further clarify optimal intervention protocols and reduce potential sources of bias, thereby strengthening the evidence for the role of Tai Chi in improving mental health among university students.

## Data Availability

The original contributions presented in the study are included in the article/Supplementary material, further inquiries can be directed to the corresponding author.
