# Supplementary material for: Effects of Tai Chi on Mental Health in college students: a systematic review and meta-analysis of randomized controlled trials
Source: Front Public Health. 2026 Jul 3;14:1830203. doi: 10.3389/fpubh.2026.1830203 (PMC13376253; doi:10.3389/fpubh.2026.1830203)

检索式：太极拳对大学生心理健康的影响：系统评价与meta分析

PubMed=49

Search: ( "Tai Ji"[Mesh] OR "tai chi"[All Fields] OR taiji[All Fields] OR taijiquan[All Fields] OR "tai chi chuan"[All Fields] OR "tai ji quan"[All Fields] ) AND ( "Students"[Mesh] OR students[All Fields] OR "college student*"[All Fields] OR "university student*"[All Fields] OR undergraduate*[All Fields] OR "graduate student*"[All Fields] ) AND ( "Mental Health"[Mesh] OR "Psychological Phenomena"[Mesh] OR "mental health"[All Fields] OR depression[All Fields] OR anxiety[All Fields] OR stress[All Fields] OR sleep[All Fields] OR "sleep quality"[All Fields] OR emotion*[All Fields] OR mood[All Fields] OR "self-esteem"[All Fields] OR "self concept"[All Fields] OR "quality of life"[All Fields] ) AND ( "randomized controlled trial"[All Fields] OR "controlled clinical trial"[All Fields] OR randomized[All Fields] OR randomised[All Fields] OR randomly[All Fields] OR trial[All Fields] )


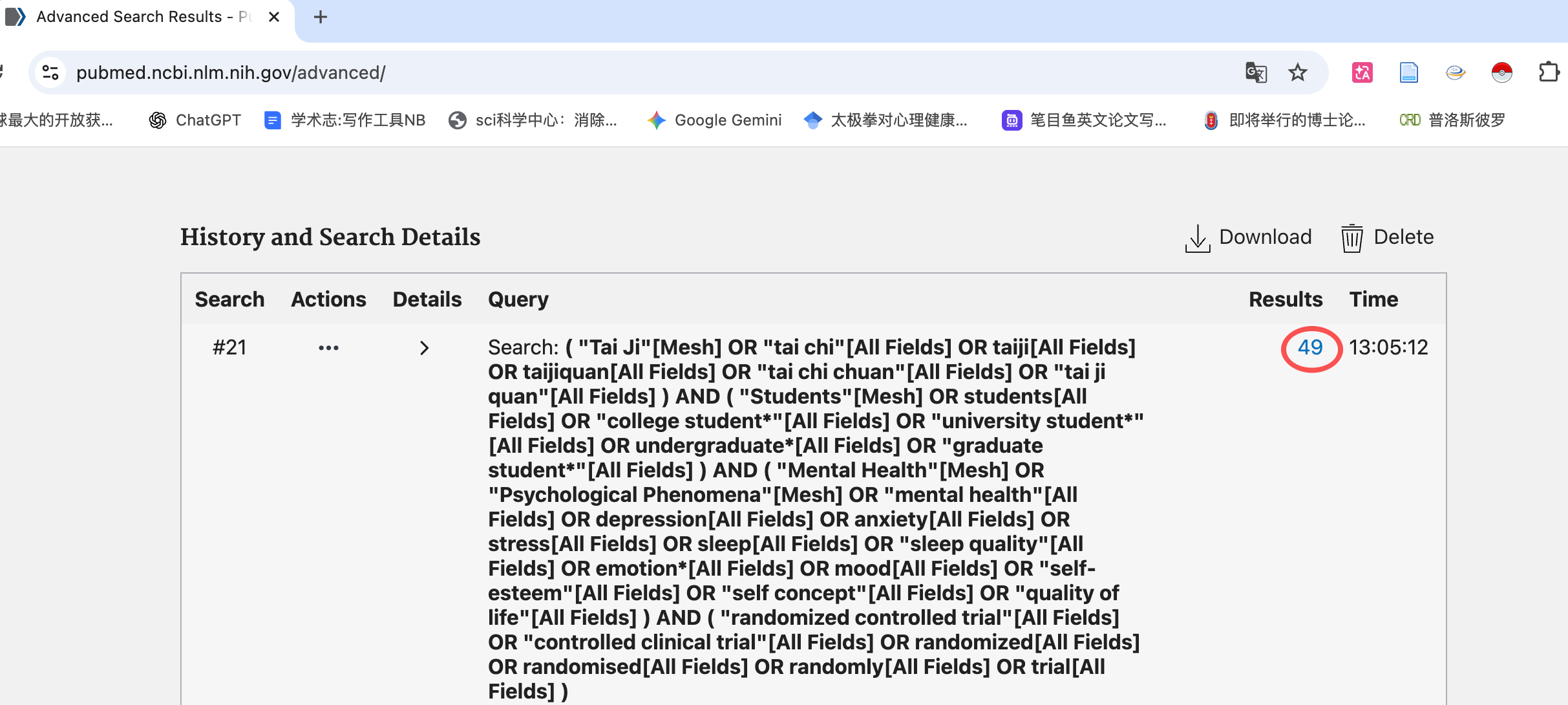


cochrane library=78

Trials matching (tai chi OR "tai chi chuan" OR taiji OR "tai ji" OR taijiquan OR "tai ji quan" OR "Chinese shadow boxing") in Title Abstract Keyword AND (student* OR university NEXT student* OR college NEXT student* OR undergraduate* OR graduate NEXT student* OR campus) in Title Abstract Keyword AND ("mental health" OR psychological OR depression OR anxiety OR stress OR sleep OR "sleep quality" OR emotion* OR mood OR "self-esteem" OR "self concept" OR "quality of life" OR wellbeing OR "well-being") in Title Abstract Keyword AND (randomized OR randomised OR randomly OR "randomized controlled trial" OR "controlled clinical trial" OR RCT OR trial) in All Text


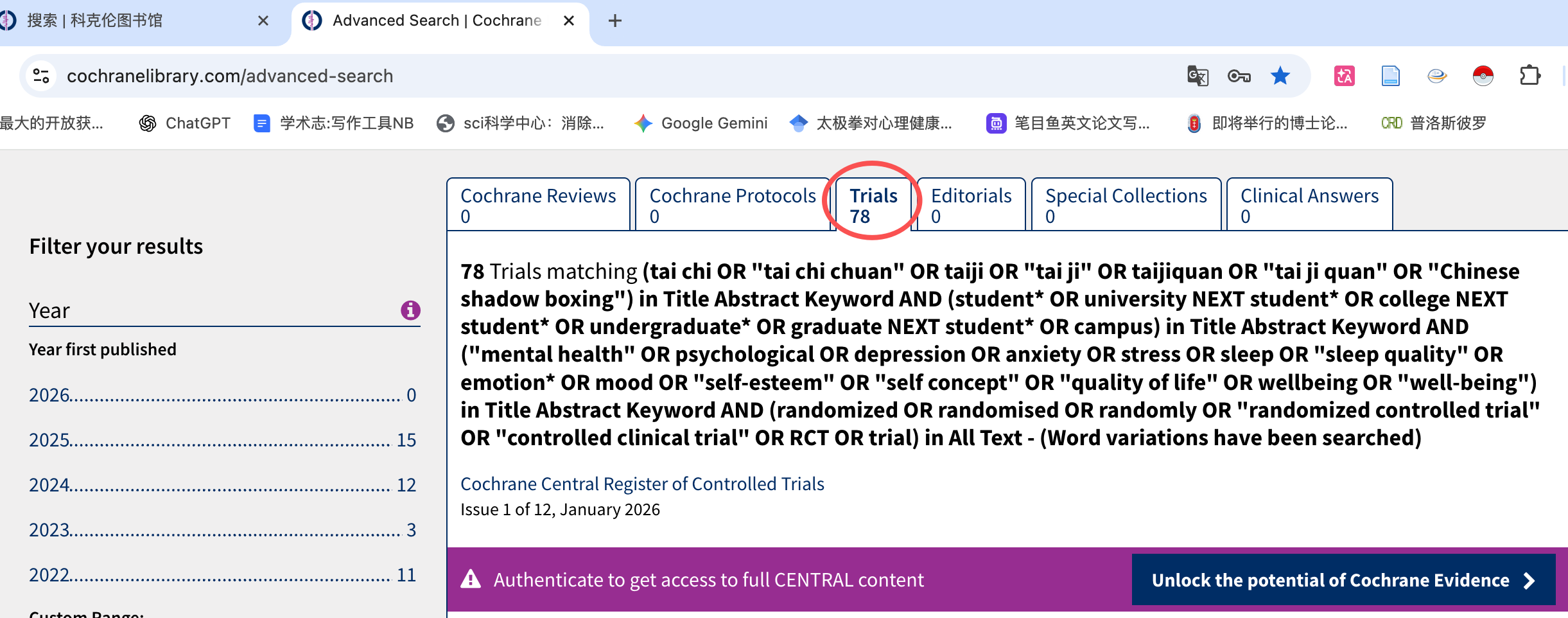


web of science=68

"tai ji" OR "tai chi" OR taiji OR taijiquan OR "tai chi chuan" OR "tai ji quan" (Topic) and students OR "college student*" OR "university student*" OR undergraduate* OR "graduate student*" (Topic) and "mental health" OR psychological OR depression OR anxiety OR stress OR sleep OR "sleep quality" OR emotion* OR mood OR "self-esteem" OR "self concept" OR "quality of life" (Topic) and "randomized controlled trial" OR "controlled clinical trial" OR randomized OR randomised OR randomly OR trial (All Fields)


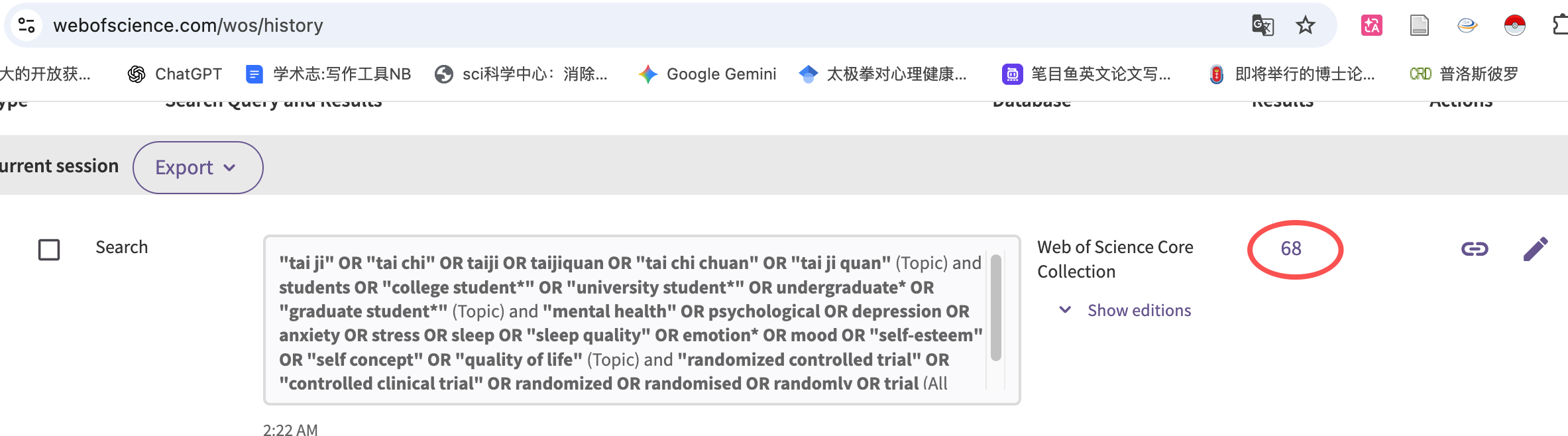


EBSCO=20

SU ("tai ji" OR "tai chi" OR taiji OR taijiquan OR "tai chi chuan" OR "tai ji quan") AND SU (students OR "college student*" OR "university student*" OR undergraduate* OR "graduate student*") AND SU ("mental health" OR psychological OR depression OR anxiety OR stress OR sleep OR "sleep quality" OR emotion* OR mood OR "self-esteem" OR "self concept" OR "quality of life") AND ("randomized controlled trial" OR "controlled clinical trial" OR randomized OR randomised OR randomly OR trial)


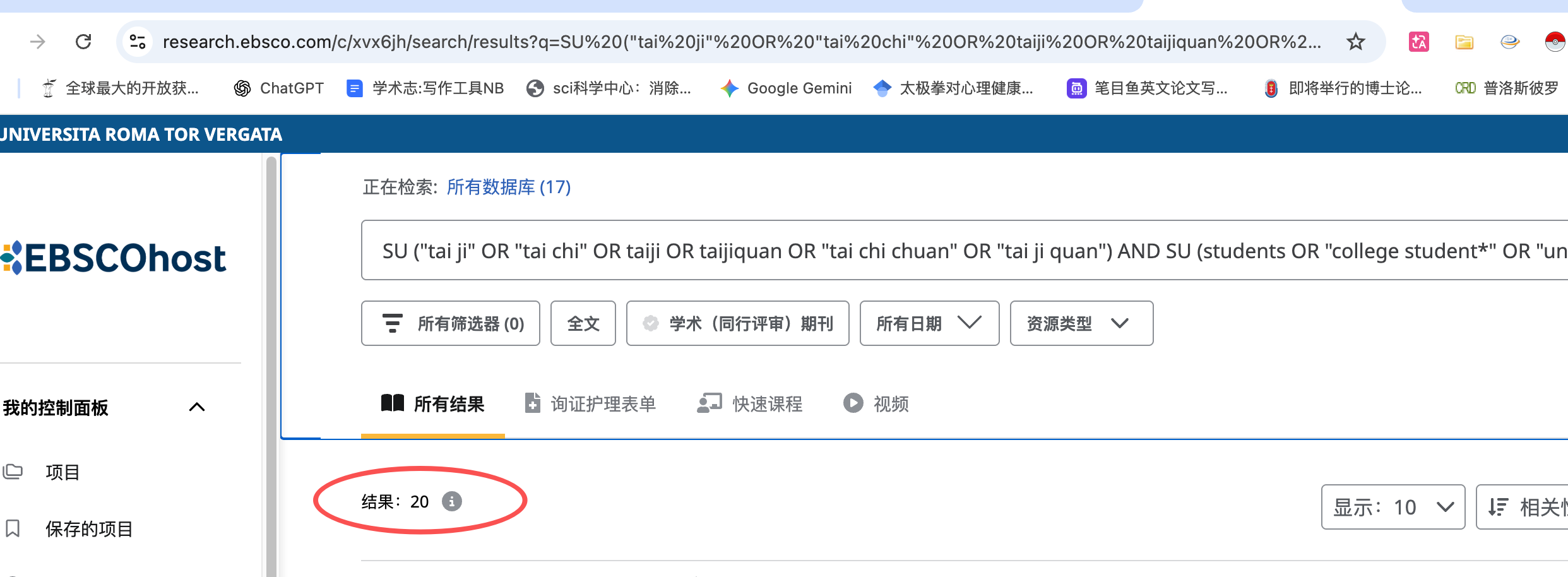


Scopus=126

TITLE-ABS-KEY ( "tai ji" OR "tai chi" OR taiji OR taijiquan OR "tai chi chuan" OR "tai ji quan" ) AND TITLE-ABS-KEY ( students OR "college student*" OR "university student*" OR undergraduate* OR "graduate student*" ) AND TITLE-ABS-KEY ( students OR "college student*" OR "university student*" OR undergraduate* OR "graduate student*" ) AND TITLE-ABS-KEY ( "mental health" OR psychological OR depression OR anxiety OR stress OR sleep OR "sleep quality" OR emotion* OR mood OR "self-esteem" OR "self concept" OR "quality of life" ) AND ALL ( "tai ji" OR "tai chi" OR taiji OR taijiquan OR "tai chi chuan" OR "tai ji quan" )


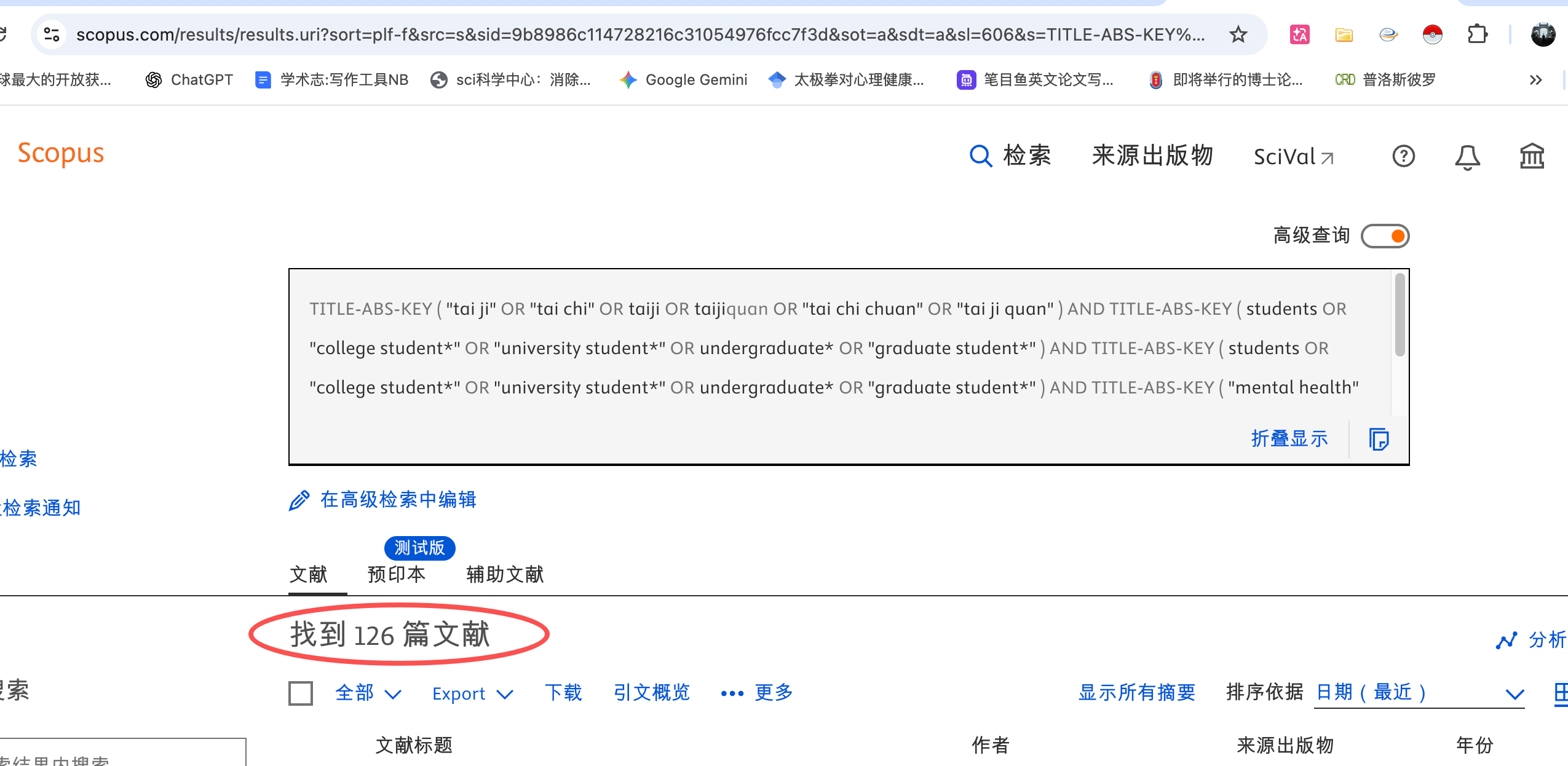


CNKI=146

（主题：太极拳）OR（主题：太极）AND（主题：大学生）OR（主题：大专生）OR（主题：本科生）OR（主题：研究生）OR（主题：高校学生）AND（主题：心理健康）OR（主题：抑郁）OR（主题：焦虑）OR（主题：压力）OR（主题：情绪）OR（主题：睡眠）OR（主题：自尊）OR（主题：自信）OR（主题：生活质量）OR（主题：心理状态）AND（全文：随机对照试验(精确)）OR（全文：实验(精确)）OR（全文：随机对照(精确)）OR（全文：随机分组(精确)）OR（全文：随机分配(精确)）


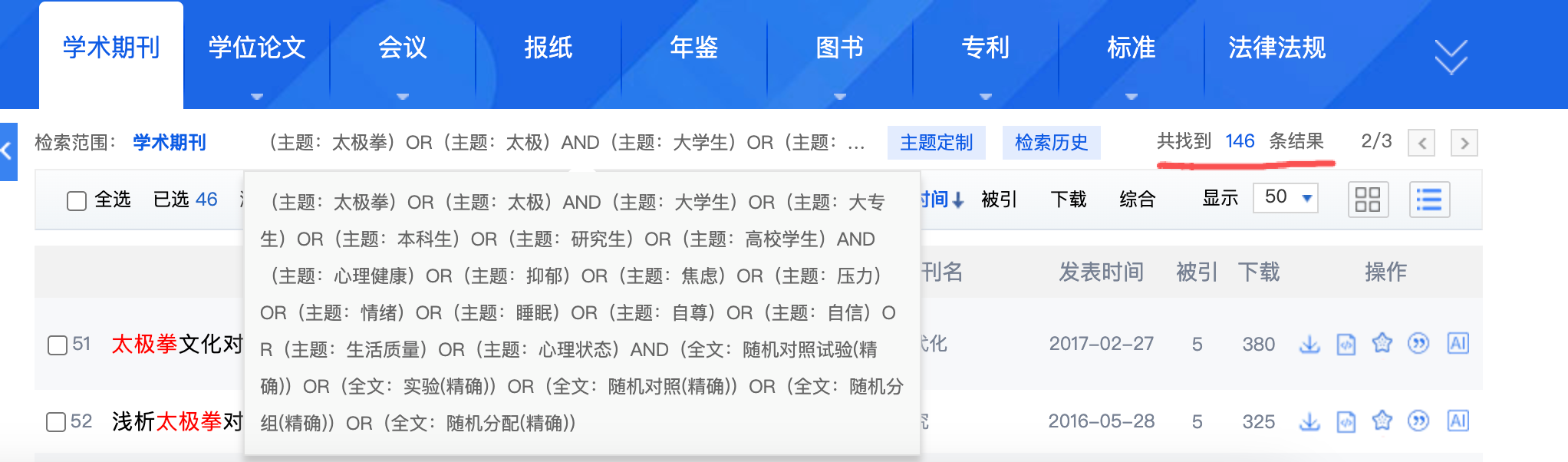


WanFang=97

主题:(太极拳 OR 太极) and 主题:(大学生 OR 大专生 OR 本科生 OR 研究生 OR 高校学生) and 主题:(心理健康 OR 抑郁 OR 焦虑 OR 压力 OR 情绪 OR 睡眠 OR 自尊 OR 自信 OR 生活质量 OR 心理状态) and 全部:(随机对照试验 OR 随机对照 OR 随机分组 OR 实验 OR 随机分配)


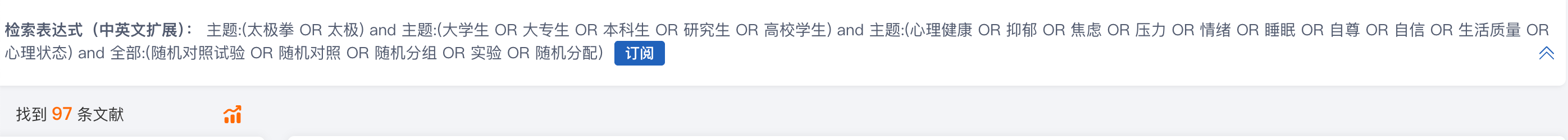


VIP=81

主题词=太极拳 OR 太极 AND 主题词=大学生 OR大专生 OR 本科生 OR 研究生 OR 高校学生 AND主题词=心理健康 OR 抑郁 OR 焦虑 OR 压力 OR 情绪 OR 睡眠 OR 自尊 OR 自信 OR 生活质量 OR 心理状态 AND 任意字段=随机对照试验 OR 随机对照OR 随机分组 OR 实验 OR 随机分配


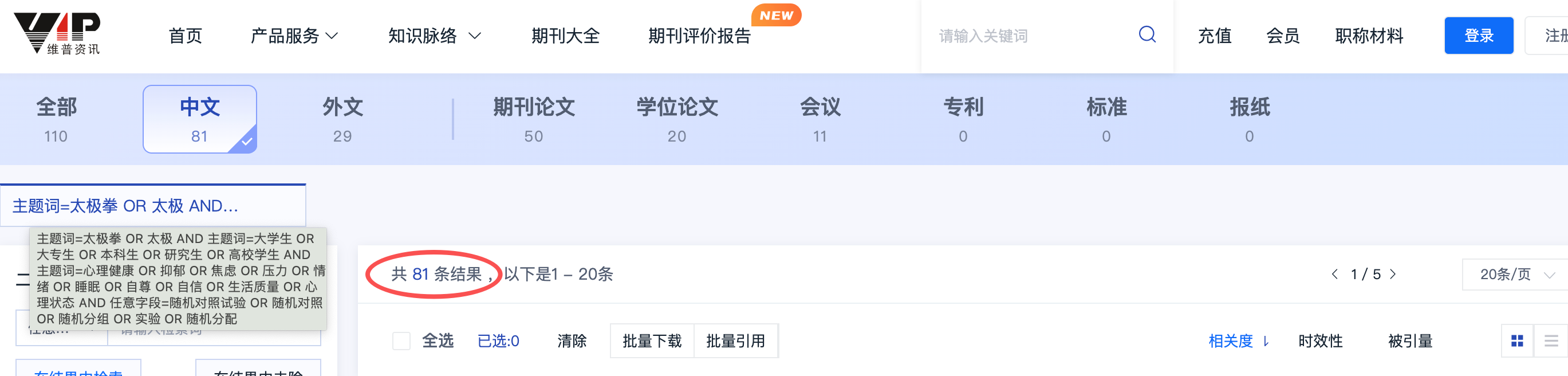

Supplement: Supplementary file 1 [file Data_Sheet_1.docx]
